# Neutrophils Inhibit Synthesis of Mineralized Extracellular Matrix by Human Bone Marrow-Derived Stromal Cells *In Vitro*

**DOI:** 10.3389/fimmu.2018.00945

**Published:** 2018-05-01

**Authors:** Okan W. Bastian, Michiel Croes, Jacqueline Alblas, Leo Koenderman, Luke P. H. Leenen, Taco J. Blokhuis

**Affiliations:** ^1^Department of Surgery, University Medical Center Utrecht, Utrecht, Netherlands; ^2^Department of Orthopaedics, University Medical Center Utrecht, Utrecht, Netherlands; ^3^Department of Respiratory Medicine, University Medical Center Utrecht, Utrecht, Netherlands; ^4^Laboratory for Translational Immunology, University Medical Center Utrecht, Utrecht, Netherlands; ^5^Department of Surgery, Maastricht University Medical Center, Maastricht, Netherlands

**Keywords:** neutrophils, bone regeneration, fracture healing, stromal cells, bone marrow stromal cell, multipotent stromal cell, alkaline phosphatase

## Abstract

Although controlled local inflammation is essential for adequate bone regeneration, several studies have shown that hyper-inflammatory conditions after major trauma are associated with impaired fracture healing. These hyper-inflammatory conditions include the trauma-induced systemic inflammatory response to major injury, open fractures, and significant injury to the surrounding soft tissues. The current literature suggests that increased or prolonged influx of neutrophils into the fracture hematoma may mediate impairment of bone regeneration after hyper-inflammatory conditions. The underlying mechanism remains unclear. We hypothesize that high neutrophil numbers inhibit synthesis of mineralized extracellular matrix (ECM) by bone marrow stromal cells (BMSCs). We therefore studied the effect of increasing concentrations of neutrophils on ECM synthesis by human BMSCs *in vitro*. Moreover, we determined how high neutrophil concentrations affect BMSC cell counts, as well as BMSC osteogenic activity determined by alkaline phosphatase (ALP) expression and ALP activity. Co-culture of BMSCs with neutrophils induced a 52% decrease in BMSC cell count (*p* < 0.01), a 64% decrease in the percentage of ALP+ cells (*p* < 0.001), a 28% decrease in total ALP activity (*p* < 0.01), and a significant decrease in the amount of mineralized ECM [38% decrease after 4 weeks (*p* < 0.05)]. Co-cultures with peripheral blood mononuclear cells and neutrophils within transwells did not induce a significant decrease in ALP activity. In conclusion, our data shows that a decreased amount of mineralized ECM became synthesized by BMSCs, when they were co-cultured with high neutrophil concentrations. Moreover, high neutrophil concentrations induced a decrease in BMSC cell counts and decreased ALP activity. Clarifying the underlying mechanism may contribute to development of therapies that augment bone regeneration or prevent impaired fracture healing after hyper-inflammatory conditions.

## Introduction

Fracture healing starts with a controlled local inflammatory response, during which inflammatory cells infiltrate the fracture hematoma (FH) that surrounds the fracture (1). It is commonly accepted that inflammatory cells not only initiate bone regeneration but are also involved in the downstream processes of fracture healing (1, 2). This is illustrated by the finding that transplantation of the early FH into muscle tissue induces ectopic bone formation (3). Moreover, removal or repeated irrigation of the early FH impairs bone healing (4, 5).

Although a controlled local inflammatory reaction seems essential for bone repair, several studies show that local and systemic hyper-inflammatory conditions are associated with impaired bone healing (1, 2). These conditions include the trauma-induced (6) systemic immune response to major injury (7, 8), open fractures (9), and significant injury to the surrounding soft tissues (10). The balance between the benefits of a controlled local inflammatory reaction on the one hand and the detrimental effects of hyper-inflammation on the other hand, suggests an optimum in the local inflammatory activity at the fracture site. In order to develop therapies that augment bone regeneration and/or prevent impairment of bone healing after hyper-inflammatory conditions, it is essential to understand how inflammatory cells influence the outcome of bone repair.

It has been shown previously that macrophages play an essential role during fracture healing (11). The numbers of anti-inflammatory M2 macrophages have been associated with increased proliferation and osteogenic differentiation of multipotent stromal cells (MSCs) *in vitro* (12) and increased bone formation in a recent animal study (11). In contrast to macrophages, only little is known about the role of neutrophils in bone healing. Our previous study showed that neutrophils contribute to fracture healing by rapidly synthesizing fibronectin+ extracellular matrix (ECM) within the human FH (13). However, animal studies suggest that high neutrophil counts within the FH are associated with impairment of fracture healing. For instance, experimental blunt chest injury, which is a model for trauma-induced damage associated molecular pattern (DAMP)-mediated systemic inflammation, induced an increased influx of neutrophils into the FH which was associated with impaired fracture healing in rats (7, 14, 15). Also, systemic depletion of neutrophils has been shown to improve the outcome of bone repair in rats (16, 17). These studies imply that high neutrophil concentrations within the FH during hyper-inflammatory conditions may negatively affect bone healing. However, the mechanism by which neutrophils affect bone regeneration remains unclear.

The inflammatory phase of fracture healing is followed by a regenerative phase, during which bone marrow stromal cells (BMSCs) and their differentiated progeny synthesize new bone tissue (18). The ECM of newly formed bone tissue mainly consists of collagen type I fibrils that become mineralized later on (18). Alkaline phosphatase (ALP) plays a crucial role in bone matrix mineralization and has, therefore, been consistently used as marker of osteogenic activity *in vivo* and *in vitro* (19).

We hypothesize that high neutrophil counts negatively affect synthesis of mineralized ECM by BMSCs. To test this hypothesis, we co-cultured human neutrophils with BMSCs and studied the effect of increasing neutrophil concentrations on ECM mineralization by BMSCs *in vitro*, as well as their effect on BMSC cell count and BMSC osteogenic activity reflected by ALP expression and ALP activity.

## Materials and Methods

### Harvesting and Isolation of BMSCs

Bone marrow stromal cells were isolated from different origins of separate donors: from patients undergoing elective orthopedic surgery of the talus (*n* = 2), patients undergoing hip arthroplasty (*n* = 3), from a 7-day-old human FH that was isolated during an open reduction internal fixation procedure (*n* = 1) and from femoral shaft reaming residues (*n* = 2), as has been described previously (20–22). Briefly, the femur was reamed using a drill-like instrument [the reamer/irrigator/aspirator (RIA), DePuy Synthes] under constant irrigation using sterile saline. The bone marrow, reaming residue, and FH were transferred to a culture facility and treated under sterile conditions. Approximately 1 g of FH and 1 g of reaming residue was divided into small fragments, resuspended in 50 ml of expansion medium (EM, Table 1), and cultured at 37°C and 5% CO_2_ in a humidified incubator. Details of all media that were used are shown in Table 1. Ficoll-Paque was used for the isolation of mononuclear cells from bone marrow aspirate of the talus and hip arthroplasty, which were subsequently seeded at a density of 0.5 × 10^6^/cm^2^.

**Table 1:** Different media used for this study.

BM	Basic Medium	α-MEM, FCS, ASAP, P/S, BGP
EM	Expansion Medium	[α-MEM, FCS, ASAP, P/S, BGP] + bFGF
OM	Osteogenic Medium	[α-MEM, FCS, ASAP, P/S, BGP] + BMP2

*Basic medium (BM) consisted of α-MEM supplemented with 10% (v/v) fetal calf serum (FCS), 0.2 mM l-ascorbic acid-2-phosphate (ASAP), 100 U/ml penicillin and 100 µg/ml streptomycin (P/S), 10 mM β-glycerophosphate (BGP). Expansion medium (EM) was BM supplemented with 1 ng/ml basic fibroblast growth factor (bFGF). Osteogenic medium was BM supplemented with 750 ng/ml rhBMP-2 (BMP2)*.

*BMP-2, bone morphogenetic protein-2*.

After 3 days of culture, bone particles and non-adherent cells were washed off the reaming residue, FH, and bone marrow-derived adherent cell population with phosphate buffered saline (PBS) twice. All subsequent washing steps were performed with PBS twice, unless mentioned otherwise. The EM was refreshed every 5 days and the adherent cells were passaged at 70–80% confluency. Subsequently, BMSCs were detached with 2 ml 0.25% trypsin-EDTA, washed in medium, and resuspended at 1.0 × 10^6^ cells/ml of fetal calf serum containing 5% (v/v) DMSO (Sigma-Aldrich) for cryopreservation at −80°C until further use.

**Figure 1:**
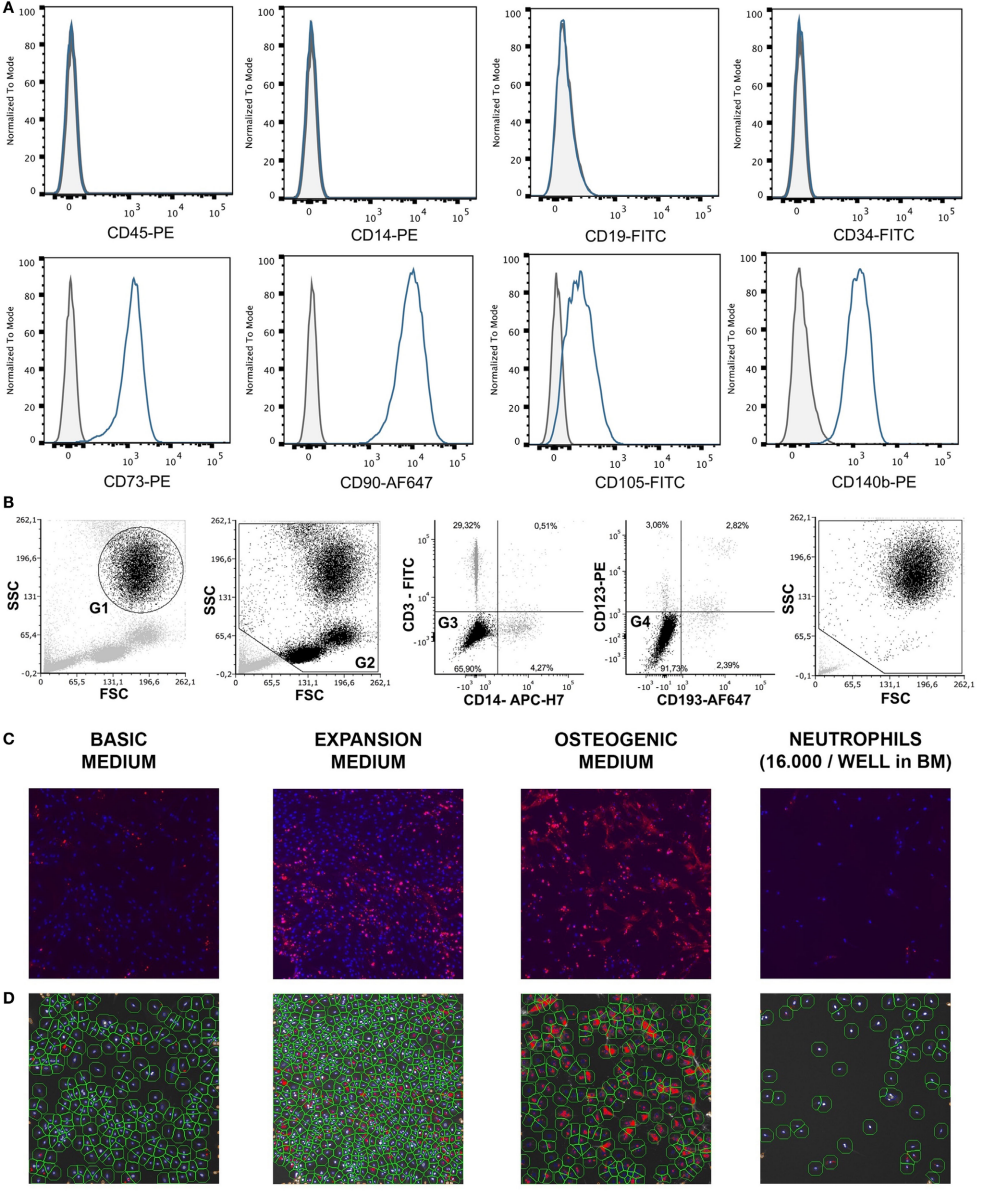
**(A)** Surface antigen expression of bone marrow stromal cells (BMSCs) isolated from the talus bone marrow using flow cytometry. >95% of cells were negative for CD45 and CD14, and >99% of cells were negative for CD19 and CD34. In addition, >95% were positive for CD73, CD90, CD105, and CD140b. Since plastic adherence is a well-established and validated technique to isolate multipotent stromal cells (MSCs), we have only characterized one BMSC donor using flowcytometry instead of all donors. The blue lines are stained cells and the gray lines are negative (unstained) controls. Adapted from Croes et al. (25). **(B)** Fluorescence-activated cell sorting (FACS) gating strategy used to isolate granulocytes/neutrophils from peripheral blood leukocytes. Granulocytes were either isolated from unlabeled leukocytes using gate 1 (G1) within the forward/sideward scatter (FSC/SSC). Alternatively, leukocytes were stained using CD3, CD14, CD193, and CD123. Within the FSC/SSC of these labeled cells, debris was first excluded [gate 2 (G2)]. Subsequently, CD3+ cells (lymphocytes) and CD14+ (monocytes) were excluded [gate 3 (G3)]. In addition, CD193+ cells (eosinophils) and CD123+ cells (basophils) were excluded [gate 4 (G4)]. The remaining CD3− CD14− CD193− CD123− cells were defined FACS-sorted neutrophils (G2+, G3+, G4+ sorted neutrophils). Re-analysis of FACS-sorted neutrophils shows adequate exclusion of lymphocytes and monocytes based on their FSC/SSC. **(C)** Images of BMSCs obtained by array scanning after 7 days of culture. BMSCs (2,000 BMSCs/well) were seeded and imaged after 7 days of culture in basic medium (BM), expansion medium (EM), osteogenic medium (OM), and after co-culture with neutrophils (16,000 neutrophils/well) in BM. Nuclei were stained with Hoechst (blue) and alkaline phosphatase (ALP) was stained (red) using Vector Red, which is a marker of osteogenic activity. All images within each experiment had similar exposure times and were not manipulated after capture with the array-scanner. **(D)** Quantification algorithm used to quantify cell count and the percentage of ALP positive cells in the adherent BMSC population after 7 days of culture. The blue rings within the algorithm images show identification of nuclei, the green rings are the area of interest around each nucleus in which Vector Red fluorescence was measured and each red pixel reflects Vector Red fluorescence above the threshold used to determine whether cells are ALP positive. The same protocol was used to quantify ALP+ cells in all experiments.

For the different assays, one cryovial containing 1.8 × 10^6^ BMSCs was rapidly thawed, diluted in basic medium (BM), centrifuged, and the cell pellet was resuspended in BM until further use. The multipotency of BMSCs isolated from the talus of one donor was established previously by standard differentiation assays along osteogenic, adipogenic, and chondrogenic lineages (23). To confirm their phenotype, these BMSCs were characterized for the expression of specific surface antigens defining human MSCs, according to the Mesenchymal and Tissue Stem Cell Committee of the ISCT (24, 25). Cells incubated for 30 min at 4°C with human FcR blocking reagent (Miltenyi, Leiden, Netherlands) and the following antibodies: CD45-PE (#560975 BD Pharmigen, Breda, Netherlands), CD14 (#R0864, Dako, Heverlee, Belgium), CD19 (130-091-328, Miltenyi, Bergisch Gladbach, Germany), CD34 (BD #555821), CD73 (BD #550257), CD90 (#B113673 BioLegend, Fell, Germany), CD105-Fitc (FAB 10971F, R&D, Minneapolis, MN, USA), and CD140b (BD #558821). After staining, cells were washed with PBS and cell fluorescence was measured in 10,000 viable cells using a BD FACSCanto II flow cytometer (Becton Dickinson, Franklin Lakes, NJ, USA). SytoxBlue (Molecular Probes/Invitrogen, Eugene, OR, USA) was used for exclusion of dead cells. >95% of cells were negative for CD45 and CD14, and >99% of cells were negative for CD19 and CD34. In addition, >95% were positive for CD73, CD90, CD105, and CD140b (Figure 1A). Previous authors have validated the plastic adherence technique used to isolate BMSCs extensively. We have, therefore, characterized only one bone marrow donor using expression of surface antigens defined by the Mesenchymal and Tissue Stem Cell Committee and differentiation assays along osteogenic, adipogenic, and chondrogenic lineages instead of characterizing all BMSC donors (24, 25). The effect of neutrophils on BMSCs was similar, regardless of BMSC source or BMSC donor.

### Isolation of Leukocytes From Peripheral Blood

Neutrophils were isolated from 12 healthy donors using two different techniques as described by us before with small modifications (26). Leukocytes were first isolated from peripheral blood of healthy volunteers by lysing erythrocytes using isotonic ice-cold ammonium chloride solution containing 155 mM NH_4_Cl, 10 mM KHCO_3_, 0.1 mM EDTA (pH 7.2). After centrifugation, leukocytes were resuspended in ice-old HEPES3+ buffer until further use. HEPES3+ contained 20 mM HEPES, 132 mM NaCl, 6 mM KCl, 1 mM MgSO_4_, 1, 2 mM KH_2_PO_4_, 1 mM CaCl_2_, 0.5% (wt/vol) human serum albumin, and 5 mM glucose. Neutrophils were either isolated from unlabeled leukocytes with fluorescence-activated cell sorting (FACS) using the granulocyte gate in the forward/sideward scatter (FSC/SSC) [Figure 1B, gate 1 (G1)]. Alternatively, leukocytes were stained using CD14-APC-H7 (BD #560180), CD3-FITC (Sony Biotechnology, #2176530), CD193 (CCR3)-AF647 (BioLegend, #310710), and CD123-PE (eBioscience, 12-1239-42). Neutrophils were defined as CD14− CD3− CD193− CD123− cells using the gating strategy as depicted in Figure 1B. Neutrophils may become activated when antibodies bind to neutrophil receptors during a positive selection technique. We, therefore, used a negative selection technique to isolate neutrophils with FACS.

Peripheral blood mononuclear cells (PBMCs) were isolated from peripheral blood of healthy volunteers using Ficoll-Paque PLUS centrifugation (GE Healthcare) for 20 min at 900 *g*. The mononuclear cell layer (ring fraction) was aspirated, centrifugated, resuspended, and stored in ice-cold HEPES3+ until further use.

### BMSC/Neutrophil Co-Cultures

Bone marrow stromal cell/neutrophil co-cultures were performed in either 96-well plate with 200 µl of medium in each well or 24-well plate with 2,000 µl of medium. Neutrophils and BMSCs were isolated from different donors (non-autologous co-cultures). In 96-well experiments, 2,000 BMSCs from the FH and reaming residues were sorted into each well using a fluorescence-activated cell sorter (MoFlo Astrios, Beckman Coulter) after excluding doublets and debris based on FSC/SSC signals. This resulted in a seeding density of 6,250 BMSCs/cm^2^. Subsequently, unlabeled granulocytes were isolated from the entire leukocyte population based on the granulocyte-specific FSC/SSC signals (gate 1, Figure 1B) (27). Either 4,000, 8,000, or 16,000 granulocytes were sorted directly into each well containing 2,000 BMSCs in 200 µl of BM. This resulted in neutrophil concentrations of 20,000, 40,000, and 80,000 neutrophils/ml, respectively. In 24-well plate experiments, BMSCs derived from the bone marrow were counted and 20,000 BMSCs were manually added to the well without the use of a sorter. This resulted in a seeding density of 10,500 BMSCs/cm^2^. Neutrophils were isolated using the FACS strategy depicted in Figure 1B (CD3− CD14− CD123− CD193− cells) and manually added to the well 160,000 neutrophils/well in 2 ml of medium (concentration of 80,000 neutrophils/ml). The cells were cultured at 37°C and 5% CO_2_ in a humidified incubator. After 3 days of culture, all media were refreshed. Since BMSCs are adherent cells and neutrophils are not, practically all neutrophils were removed from the co-culture at this time point. Subsequently, no new neutrophils were added to the monolayer of BMSCs and the media were refreshed approximately every 3–4 days. After 7 days, BMSC cell counts and osteogenic activity of BMSCs was quantified as described below. After 4 weeks of culture, mineralization of ECM was quantified. Each condition was established in duplicates. The experiments were repeated with neutrophils and BMSCs from different donors and the experiments were set up at different dates.

### Trans-Well Experiments

To determine whether an effect of neutrophils on BMSC osteogenic activity was dependent on soluble factors, transwell experiments were performed. The highest neutrophil concentration (80,000 neutrophils/ml) was compared to BMSC monocultures. The use of transwell-inserts required usage of 24-well plate instead of 96-well. Since the total volume of a 24-well plate well is approximately 10 times the volume of a 96-well plate well, the number of BMSCs and neutrophils per well were adjusted accordingly without altering the ratio between neutrophils and BMSCs and the concentration of neutrophils/ml. Each well within the 24-well plate contained 2 ml of medium, seeded with 20,000 BMSCs and 160,000 neutrophils.

### Analysis of ALP Activity

For quantitative ALP determination, cells were lysed in 0.2% (v/v) Triton X-100 in PBS for 30 min. ALP activity was measured by conversion of the p-nitrophenyl phosphate Liquid Substrate System (Sigma-Aldrich). The absorbance was measured at 405 nm and corrected at 655 nm on a multi-well plate reader (Bio-Rad, Hercules, CA, USA). Values were normalized to ALP activity in BMSCs cultured in BM.

### Analysis of BMSC Cell Count and ALP Expression

To determine whether a decreased ALP activity was caused by a decreased number of ALP+ cells or a decreased expression of ALP on ALP+ cells, the adherent cell population was stained and imaged using an array-scanner. After 7 days of co-culture, adherent cells were washed, fixed with 3.7% neutral buffered formaldehyde solution for 10 min at room temperature, washed and incubated with Vector Red alkaline phosphatase substrate kit (Vector Labs) for 1 h at room temperature in the dark. The Vector Red solution was prepared according to the manufacturer’s protocol. Subsequently, adherent cells were washed and stained with Hoechst 33258 (Sigma-Aldrich) 10 µg/ml in PBS for 30 min at room temperature in the dark, washed, and stored in PBS until further analysis. BMSCs were counted by quantifying the number of nuclei within six microscopy fields with an array-scanner (ArrayScan VTI HCS Reader, Thermo Scientific). The nuclei were identified based on Hoechst staining, size and shape of the nucleus (Figures 1C,D). All nuclei at the image borders were excluded from the analysis. The first microscopy field was the standardized center of each well and every following microscopy field followed a standardized automated spiral track toward the periphery of the well. Osteogenic activity was measured by placing a standardized ring-shaped area of interest around each nucleus and measuring fluorescence of Vector Red within this area of interest using the manufacturer’s Spot Detection protocol^1^ (Figure 1D). This technique allowed measurement of ALP expression for each individual BMSC. In brief, the Spot Detection protocol places a grid of 512 × 512 pixels over each grayscale array-scanner image. Each pixel within the image has a brightness-value ranging from black to white. The brightness of each pixel, therefore, corresponds with the amount of fluorescence. After setting a threshold, each pixel within the grid becomes either positive or negative (positive pixels become stained red in Figure 1D). The Spot Detection protocol counts the number of positive pixels within each area of interest around each nucleus (green circles in Figure 1D). The determination of the area of interest was standardized as described in the manufacturer’s Spot Detection protocol (see text footnote 1). An additional threshold makes each cell either positive or negative based on the number of positive spots within the area of interest. The exposure time was set, based on the first well containing BMSCs in BM without neutrophils. The exposure time remained identical for all conditions within an experiment. The threshold remained identical within and between experiments. The exposure time may vary between experiments, for instance based on the time between staining of BMSCs and the eventual time of imaging. ALP is crucial for ECM mineralization *in vivo* and *in vitro* and is, therefore, a well-established marker of osteogenic activity (28, 29).

### Analysis of ECM Mineralization Using Alizarin Red

**Figure 2:**
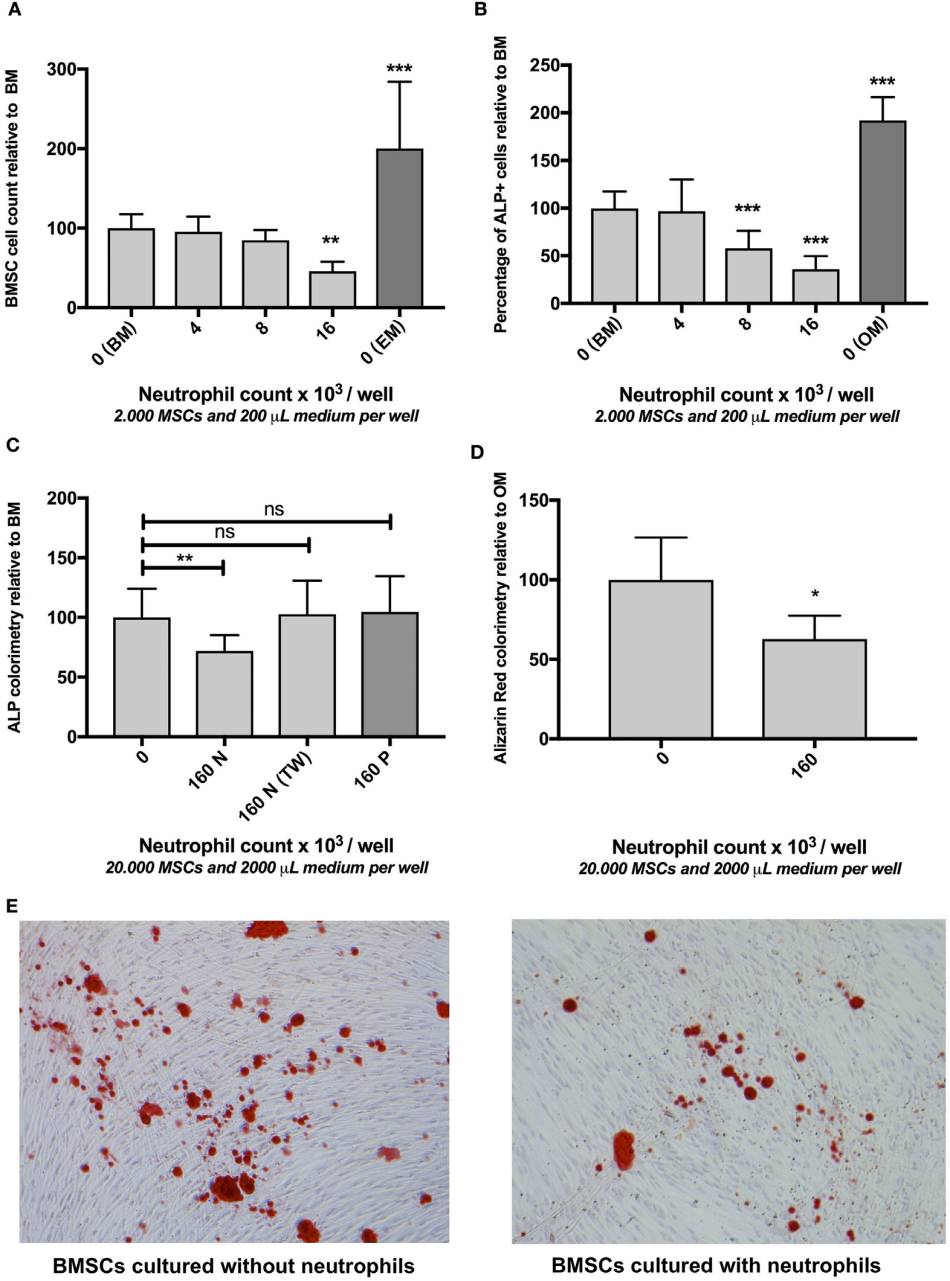
**(A)** The effect of neutrophils on bone marrow stromal cells (BMSCs) cell count *in vitro* (mean ± SEM/6 microscopy fields). Co-culture of BMSCs with different neutrophil concentrations resulted in decreased BMSC counts after 7 days of culture. Neutrophils were isolated from unlabeled leukocytes based on granulocyte-specific forward/sideward scatter (FSC/SSC) (Figure 1B) from three donors and cultured with three different BMSC donors [reamer/irrigator/aspirator (RIA) (*n* = 2) and fracture hematoma (FH) (*n* = 1)] in a 96-well plate ***p < 0.001, **p < 0.01 compared to BMSCs cultured in BM without neutrophils. BMSCs cultured without neutrophils in expansion medium (EM) are illustrated by the dark gray bar. **(B)** The effect of neutrophils on osteogenic activity of BMSCs *in vitro* (mean ± SEM/6 microscopy fields). Co-culture with different neutrophil concentrations induced a decreased percentage of alkaline phosphatase (ALP) positive cells after 7 days of culture. The same cells and number of donors were used as described in panel **(A)**. The percentage of ALP+ FH and RIA-derived BMSC was 32 and 29%, respectively (cultured without neutrophils). FH- and RIA-derived BMSCs cultured without neutrophils were pooled (BM). All other conditions are depicted relative to BM. Therefore, the mean percentage of ALP+ of BM was set to 100%. BMSCs cultured without neutrophils in OM are illustrated by the dark gray bar.***p < 0.001 compared to BMSCs cultured in BM without neutrophils. **(C)** The effect of fluorescence-activated cell sorting (FACS) sorted neutrophils, peripheral blood mononuclear cells (PBMCs), and neutrophil transwell co-culture on osteogenic activity of BMSCs *in vitro* after 1 week of culture (mean ± SEM). FACS-sorted CD3− CD14− CD123− CD193− neutrophils (three donors, Figure 1B) were co-cultured with bone marrow-derived BMSCs (two donors) in a 24-well plate containing basic medium (BM), which induced a significant decrease in osteogenic activity (160N = 160,000 neutrophils/well). By contrast, Ficoll isolated PBMCs did not induce a significant decrease in ALP activity (160P = 160,000 PBMCs/well in BM). Moreover, transwell experiments in which neutrophils and BMSCs did not have cell–cell contact also did not significantly inhibit osteogenic activity [160N (TW) = 160,000 neutrophils/transwell insert in BM]. **(D)** The effect of FACS sorted neutrophils on extracellular matrix (ECM) *in vitro* after 4 weeks of culture (mean ± SEM). FACS sorted neutrophil co-culture with BMSCs in osteogenic medium (OM) induced a significant decrease in ECM mineralization after 4 weeks of culture as analyzed by Alizarin Red staining compared to BMSCs that were cultured in OM alone. *p < 0.05. **(E)** Representative images of Alizarin Red stained monolayers of BMSCs after 4 weeks of culture with and without neutrophils.

After 4 weeks of culture in osteogenic medium (OM), the adherent cell population was washed with PBS and fixed in 4% (w/v) paraformaldehyde, stained for 10 min with 2% (w/v) Alizarin Red solution (pH 4.2, Sigma-Aldrich) and examined by light microscopy (Figure 2E). In addition, Alizarin Red was extracted from the monolayer by incubating the adherent cells in 1.0 ml 10% cetylpyridinium chloride buffer for 30 min. The dye was dissolved in the well and 200 µl aliquots were transferred to a 96-well plate prior to reading at 595 nm. The data were corrected by subtraction of a background reading at 655 nm.

### CFSE Labeling of Neutrophils

**Figure 3:**
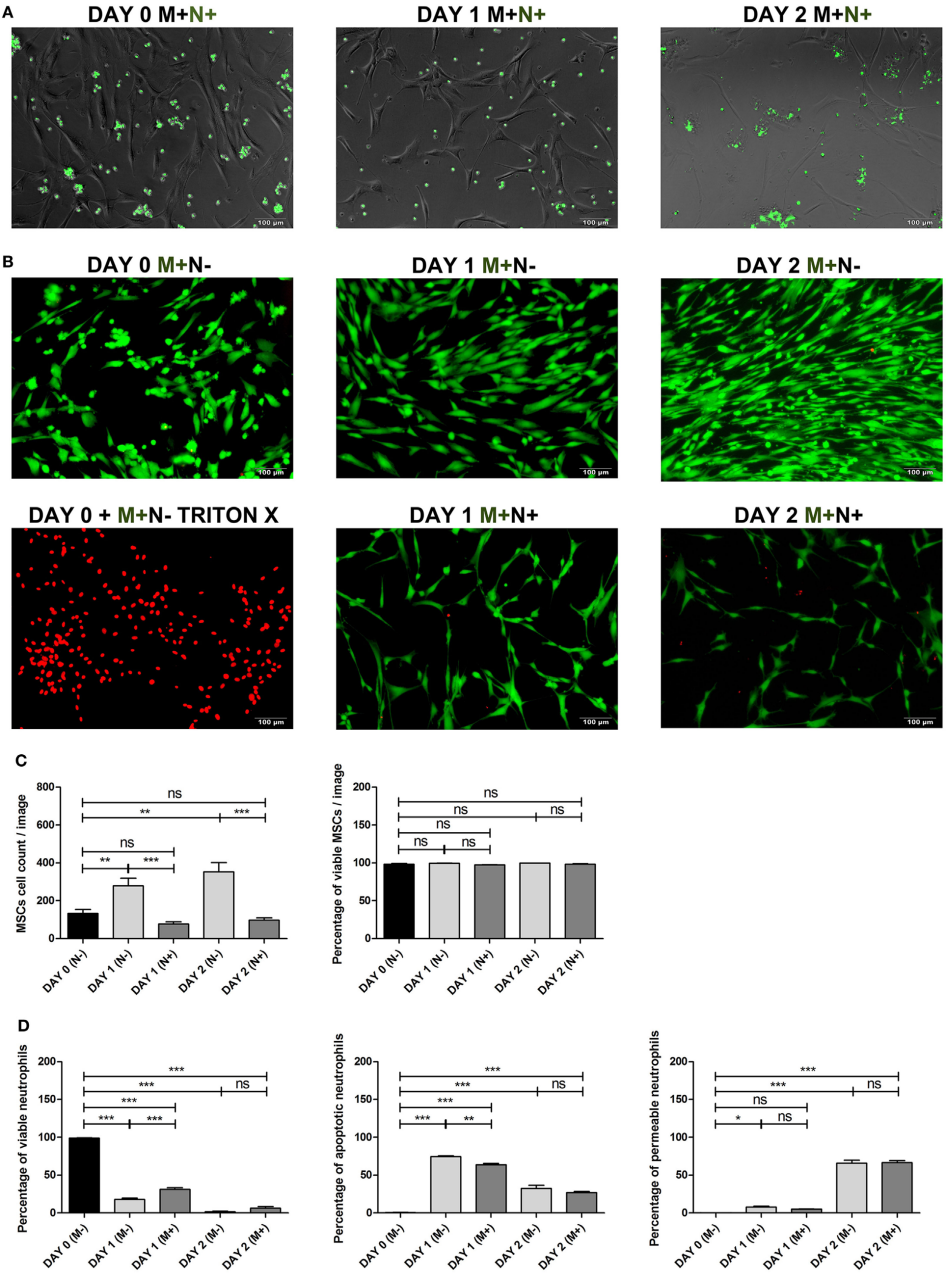
**(A)** Bone marrow stromal cell (BMSC) co-cultured with CFSE labeled neutrophils. BMSCs were co-cultured with CFSE-labeled neutrophils (green) and imaged using fluorescence microscopy at day 0 and after 1 and 2 days of culture with BMSCs to determine whether BMSCs phagocytosize neutrophils. BMSCs did not become CFSE positive after co-culture with CFSE labeled neutrophils. This finding suggests that phagocytosis of (apoptotic) neutrophils by BMSCs *in vitro* is not likely. **(B)** BMSCs LIVE/DEAD™ assay. Representative images of BMSCs stained with a LIVE/DEAD™ kit before and after culture with and without neutrophils. Viable BMSCs are green and non-viable BMSCs are red. Triton-X killed BMSCs were used as positive control. BMSCs (M+) were imaged on day 0, day 1, and day 2, cultured with (N+) or without (N−) neutrophils. **(C)** BMSCs viability and BMSC cell count (mean ± SEM). BMSC was stained with the LIVE/DEAD™ kit before and after 1 and 2 days of culture with and without neutrophils. Three neutrophil donors and two BMSC donors (two arthroplasty) were used (*n* = 6 conditions). The total BMSC count per microscopy image and the percentage of viable (green) BMSCs is depicted. BMSC counts increased during the first 2 days of culture without neutrophils and practically all cells remained viable (green). By contrast, the number of BMSCs decreased during the first 2 days after co-culture with neutrophils. The percentage of viable BMSCs did not significantly differ at day 1 and day 2 when cultures with and without neutrophils were compared. ***p < 0.001, **p < 0.01, *p < 0.05; ns, not significant. **(D)** Neutrophil viability assay. Neutrophils were stained with Annexin and 7-AAD before and after 1 and 2 days of culture with and without BMSCs. Three neutrophil donors and four BMSCs donors (one talus and three arthroplasty) were used (*n* = 12 conditions). The graphs depict the percentage of viable cells (Annexin and 7-AAD double negative cells), apoptotic cells (Annexin positive, 7-AAD negative), and permeable cells (Annexin and 7-AAD double positive cells). As has been described previously, we found that BMSCs induced a significant increase in the percentage of viable neutrophils at day 1 of culture. However, after 2 days of culture, practically all neutrophils were non-viable and there was no significant difference between neutrophils cultured with and without BMSCs at day 2. The median fluorescence of Annexin and 7-AAD was pooled and depicted as mean ± SEM. ***p < 0.001, **p < 0.01, *p < 0.05; ns, not significant.

It has been shown previously that phagocytosis of apoptotic cells influences BMSC osteogenic differentiation (30). We have therefore stained neutrophils with CFSE to determine whether BMSCs phagocytize (apoptotic) neutrophils. The membrane permeable carboxyfluorescein diacetate succinimidyl ester (CFDA-SE) is converted to the fluorescent membrane-impermeable CFSE by intracellular esterases after which the fluorescent CFSE covalently couples to intracellular molecules. Neutrophils were washed with serum-free RPMI medium (Gibco) and resuspended in RPMI at a concentration of 1 × 10^6^ neutrophils/ml. Subsequently, neutrophils were labeled with CFDA-SE (Sigma-Aldrich) by diluting the 5 mM stock 1:200 and neutrophils were incubated for 5 min at room temperature. Subsequently, neutrophils were washed in HEPES3+ twice and resuspended in BM. The fluorescently labeled neutrophils were imaged at day 0, day 1, and day 2 after co-culture with BMSCs using an Olympus XI53 fluorescent microscope (Figure 3A).

### BMSC Viability Assay and BMSC Cell Counts

The effect of neutrophil co-culture on BMSC viability was tested using the Molecular Probes™ LIVE/DEAD™ assay (ThermoFisher Scientific) according to the manufacturer’s protocol.^2^ Viable cells were fluorescently labeled green and non-viable cells were fluorescently labeled red. Triton-X killed BMSCs were used as a positive control. BMSCs were imaged on day 0, day 1, and day 2 with and without neutrophils using an Olympus XI53 fluorescent microscope (Figure 3B). Total BMSC counts and percentage viable cells were manually counted and depicted in Figure 3C.

### Neutrophil Viability Assay

The effect of BMSC co-culture on neutrophil viability was assessed using the PE Annexin V Apoptosis Detection Kit which also contains 7-AAD staining (BD Pharmingen) according to the manufacturer’s protocol.^3^ Viable cells with intact membranes exclude 7-AAD, whereas the membranes of dead and damaged cells are permeable to 7-AAD. Annexin V staining precedes the loss of membrane integrity, which allows identification of early apoptotic cells (7-AAD negative, PE Annexin V positive). Neutrophil viability was assessed before co-culture at day 0, after 1 and 2 days of culture with and without BMSCs. Figure 3D shows the percentage of viable neutrophils (Annexin and 7-AAD double negative cells), the percentage apoptotic neutrophils (Annexin positive, 7-AAD negative), and the percentage of permeable neutrophils (Annexin and 7-AAD double positive cells) for abovementioned conditions.

### Expression of Surface Markers on Neutrophils

**Figure 4:**
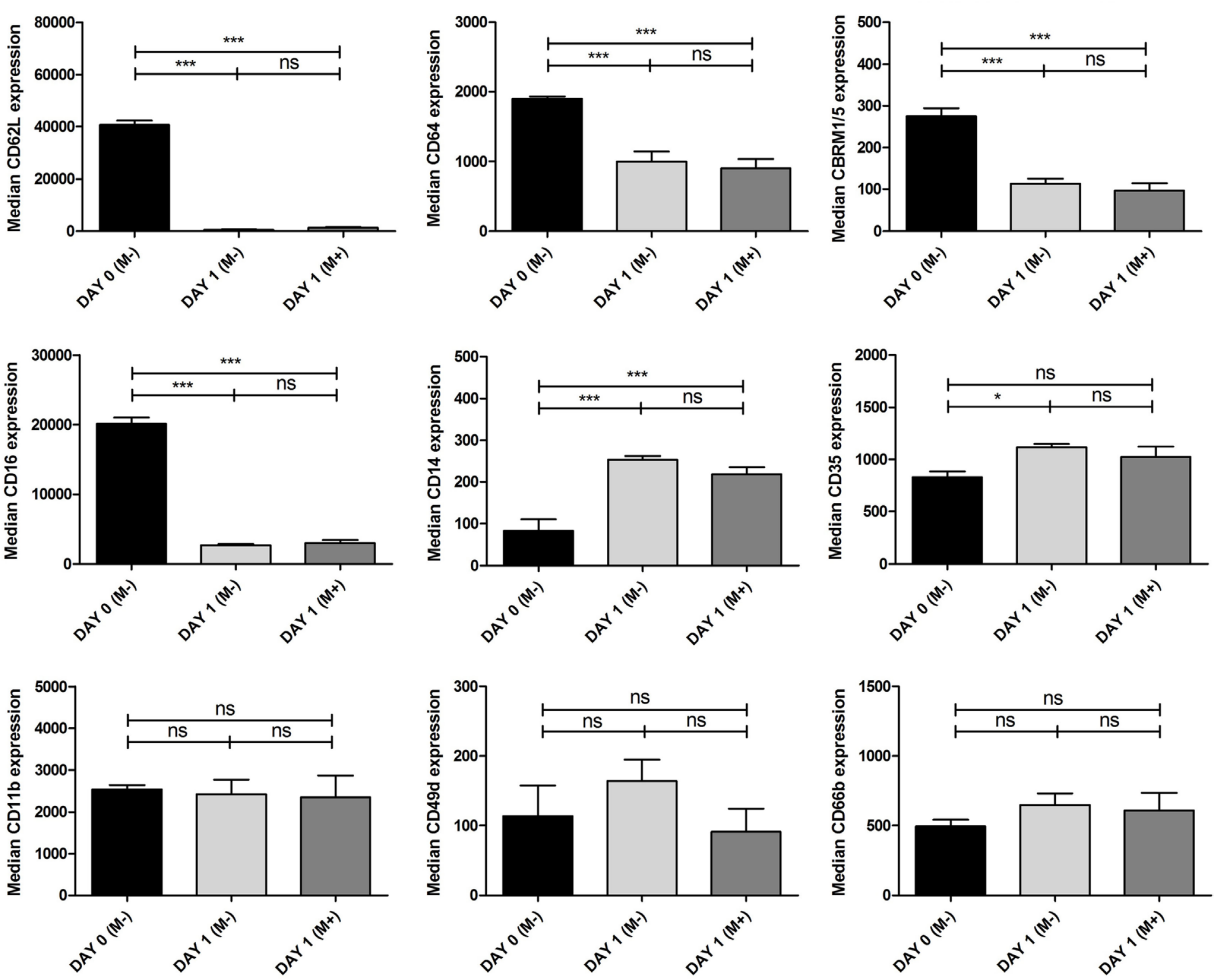
Expression of surface markers on neutrophils. Multicolor flow cytometry was used to quantify expression of CD62L, CD64, CBRM1/5, CD16, CD14, CD35, CD11b, CD49d, and CD 66b on neutrophils before and after 24 h culture with and without bone marrow stromal cells (BMSCs) in BM. Neutrophils were isolated from three donors and cultured with BMSCs isolated from four donors (one talus and three arthroplasty) (*n* = 12 combinations). The black bars represent uncultured neutrophils and therefore without BMSCs [day 0 (M−)]. The gray bars represent neutrophils cultured for 24 h with [day 1 (M+), dark gray] or without [day 1 (M−), light gray] BMSCs in BM. The median fluorescence of each surface marker was pooled and depicted as mean ± SEM. ***p < 0.001, **p < 0.01, *p < 0.05; ns, not significant.

The effect of neutrophil culture with and without BMSC on expression of neutrophil surface markers associated with an activated phenotype (31–34) was assessed using multicolor flowcytometry. Neutrophils were washed with PBS containing sodium citrate 0.32% and albumin 4 g/l (PBS^2+^) and resuspended in a solution containing CD35-FITC (E11), CD66b-PerCP-Cy5.5 (G10F5), CD49d-PeCy7 (9F10), CD64-AF647, CBRM1/5-AF700, and CD11b-AF750 from BioLegend, CD62L-BV650 from BD Biosciences, CD14-e450 from ThermoFisher Scientific and CD16-Krome Orange (3G8) from Beckman Coulter in PBS^2+^. After incubation with the antibody solution for 30 min on ice, neutrophils were washed with PBS^2+^ and analyzed using a BD LSRFortessa™ cell analyzer (Becton Dickinson, Mountain View, CA, USA). Neutrophils were identified according to their specific forward-/side-scatter patterns. Flow cytometry data were analyzed with FlowJo^®^ v10 software (FlowJo, LLC, Ashland, OR, USA). The median fluorescence of each marker before and after culture with and without BMSCs after 24 h is depicted in Figure 4.

### Statistical Analysis

Based on whether the data was normally distributed, an ANOVA or Kruskal–Wallis analysis was used to compare groups. Each experimental condition was compared to the control condition (medium alone) with a Dunnet *post hoc* test or Mann–Whitney *U* using Bonferroni’s correction for multiple testing (two-tailed). All data are presented as mean ± SEM compared to the mean of all control conditions together (BMSC monocultures in medium, unless indicated otherwise). The mean of all control conditions together was therefore 100%. The statistical analyses were performed using GraphPad Prism version 5.03 (GraphPad Software, Inc.). A *p*-value <0.05 was considered to be statistically significant. The data generated during this study are available from the corresponding author on reasonable request. BMSCs were isolated from residual samples and therefore collected without informed consent, unless the patient refused explicitly (opt-out method). All samples were acquired in accordance with relevant guidelines and regulations.

### Ethics Statement

The medical-ethical committee of the University Medical Center of Utrecht has approved isolation and use of residual samples after anonymization without informed consent. Leukocytes from peripheral blood of healthy donors were acquired after informed consent. The medical-ethical committee of the University Medical Center of Utrecht also approved isolation and use of peripheral blood of healthy donors after informed consent and anonymization.

## Results

### The Effect of Neutrophils on BMSC Cell Count *In Vitro*

As illustrated in Figure 2A, co-culture of BMSCs with high neutrophil concentrations induced a 54% decrease in the number of BMSCs after 7 days of culture compared to monocultures of BMSCs in BM (100%, *n* = 9, *p* < 0.01). Culture of BMSCs in 200 µl of EM for 7 days induced a 100% increase in BMSCs cell counts compared to BMSCs that were cultured in BM for 1 week (100%, *n* = 9, *p* < 0.001).

### The Effect of Neutrophils on Osteogenic Activity of BMSCs *In Vitro*

Subsequently the effect of neutrophil co-culture was studied on the percentage of ALP+ BMSCs as well as total ALP activity within the entire BMSC population. Co-culture of BMSCs with high neutrophil concentrations caused a 64% decrease in the percentage of ALP+ cells within the entire BMSC population after 7 days of culture compared to monocultures of BMSCs (100%, *n* = 9, *p* < 0.001, Figure 2B). Culture of BMSCs in OM for 1 week induced a 92% increase in the percentage of ALP+ cells compared to BMSCs cultured in BM for 1 week (100%, *n* = 9, *p* < 0.001). FACS-sorted CD3− CD14− CD123− CD193− neutrophils (Figure 1B) were co-cultured with bone marrow-derived BMSCs (Figure 1A) for 1 week and induced a 28% decrease in ALP activity (*p* < 0.01, *n* = 8, Figure 2C). Co-culture of these BMSCs with Ficoll isolated PBMCs did not induce a significant decrease in ALP activity (*n* = 8, Figure 2C). Moreover, FACS-sorted neutrophils co-cultured with transwell also did not induce a significant decrease in ALP activity (*n* = 6, Figure 2C).

### The Effect of Neutrophils on ECM Mineralization *In Vitro*

FACS-sorted CD3− CD14− CD123− CD193− neutrophils (Figure 1B) were co-cultured with bone marrow-derived BMSCs (Figure 1A) and induced a 38% decrease in mineralized ECM after 4 weeks of culture in OM analyzed by Alizarin Red staining (*p* < 0.05, *n* = 4, Figures 2D,E).

### BMSC Co-Cultured With CFSE Labeled Neutrophils

It has been shown previously that phagocytosis of apoptotic cells influences BMSC osteogenic differentiation (30). We have, therefore, stained neutrophils with CFSE to determine whether BMSCs phagocytize (apoptotic) neutrophils. As shown in Figure 3A, BMSCs did not become CFSE positive after co-culture with CFSE labeled neutrophils. This finding suggests that phagocytosis of (apoptotic) neutrophils by BMSCs *in vitro* is not likely.

### BMSCs Viability After Culture With and Without Neutrophils

We determined whether a decreased BMSC counts after neutrophil co-culture (Figure 2A) may be caused by neutrophil induced cell death of BMSC. As depicted in Figures 3B,C, BMSC counts increased during the first 2 days of culture without neutrophils and practically all cells were viable (green). By contrast, the number of BMSCs decreased during the first 2 days after co-culture with neutrophils. The percentage of viable BMSCs did not significantly differ at day 1 and day 2 when cultures with and without neutrophils were compared (Figure 3C).

### Neutrophil Viability After Culture With and Without BMSCs

It has been shown previously that BMSCs increase neutrophil survival *in vitro* (35). We determined neutrophil viability before and after culture with and without BMSCs. Figure 3D shows that the percentage of viable cells (Annexin and 7-AAD double negative cells) significantly decreases during the first 2 days of culture. As has been described previously, we found that BMSCs induced a significant increase in the percentage of viable neutrophils at day 1 of culture. However, after 2 days of culture, practically all neutrophils were non-viable and there was no significant difference between neutrophils cultured with and without BMSCs at day 2.

### Expression of Surface Markers on Neutrophils

To determine whether BMSCs affect expression of surface markers on neutrophils associated with an activated phenotype, we used multicolor flow cytometry to quantify expression of CD62L, CD64, CBRM1/5, CD16, CD14, CD35, CD11b, CD49d, and CD 66b on neutrophils. We found that uncultured neutrophils had significantly higher expression of CD62L, CD64, CBRM 1/5, and CD16 and lower expression of CD14 compared to neutrophils that were cultured with or without BMSCs. There was no significant difference in expression of CD11b, CD49d, and CD66b between uncultured and cultured neutrophils. We did not find a significant difference in expression of surface markers between neutrophils that were cultured with and without BMSCs after 24 h of culture.

## Discussion

Our study shows that human neutrophils can induce a significant decrease in the amount of ECM that becomes synthesized by BMSCs *in vitro* (Figures 2D,E). Moreover, our data indicate that neutrophils induce a significant decrease in BMSC cell count, percentage of ALP positive cells, and total ALP activity, which is a marker of osteogenic activity (Figures 2A–C). Neutrophils that were co-cultured in transwells, which prevented cell–cell contact between neutrophils and BMSCs, did not induce a significant decrease in ALP activity (Figure 2C). Moreover, PBMCs also did not induce a significant decrease in ALP activity (Figure 2C). Our human data are in line with animal studies which have shown that systemic depletion of neutrophils improves bone healing and induces increased expression of osteogenic transcription factors at the site of bone injury (16, 17).

It has been shown previously that peripheral blood leukocytes can inhibit growth of bone marrow-derived stromal cells *in vitro* (36). Our finding that neutrophils inhibit synthesis of mineralized ECM by BMSCs *in vitro* may be part of a physiological regenerative process. The early FH contains a significant amount of debris that needs to become degraded and phagocytized by inflammatory cells, which are known to be capable of releasing reactive oxygen species (ROS) and ECM degrading enzymes (1, 2, 37, 38). Moreover, the FH may be contaminated by pathogens in case of open fractures that also need to become neutralized by inflammatory cells. It is plausible that these processes make the early FH a suboptimal environment for BMSCs to start synthesizing new bone ECM. Our previous study showed that neutrophils infiltrate the human FH within 12 h after injury and contribute to fracture healing by synthesizing fibronectin+ ECM (13). A recent animal study also suggested a beneficial effect of neutrophils during early bone regeneration (39). At day 3–5 after injury, neutrophil concentrations decrease after which the first BMSCs become apparent within the human FH and BMSCs numbers start to increase (13). We hypothesize that neutrophils keep BMSCs in “stand-by mode” by inhibiting their proliferation and differentiation until debridement of the FH, neutralization of pathogens and synthesis of fibronectin+ ECM has sufficiently been accomplished. Although this effect of neutrophils on BMSCs may be physiological, we speculate that this effect can negatively affect the outcome of bone healing during or after hyper-inflammatory conditions. Based on the abovementioned study (13), we believe that the ratios between neutrophils and BMSCs used in our *in vitro* co-cultures may be similar to the *in vivo* ratios.

Our recent study showed that multitrauma patients who develop impaired bone healing have decreased peripheral blood neutrophil concentrations during the first 2 weeks after injury, which may be explained by increased extravasation of neutrophils (8). It is known that severe injury causes release of DAMPs (6) into the peripheral circulation, which induces a systemic inflammatory response (40). Neutrophils can become primed or pre-activated during trauma-induced systemic inflammation and exhibit enhanced migration toward inflammatory stimuli, such as the FH (7). Our finding that neutrophils inhibit synthesis of mineralized ECM by BMSCs may explain how increased or prolonged influx of neutrophils into the FH disturbs the regenerative phase of fracture healing after major trauma.

Neutrophils are short lived, especially on plastic surfaces in vitro (41), and it has been shown previously that BMSCs induce prolonged survival of neutrophil *in vitro* (35). In our co-culture, practically all neutrophils were non-viable after 2 days of culture, either with or without BMSCs (Figure 3C). These neutrophils were washed off the monolayer of adherent BMSCs at day 3, when the culture medium is refreshed. It is, therefore, likely that neutrophils exert their effect on BMSCs within the first 3 days of co-culture. It is well known that neutrophils are equipped with an extensive cytotoxic armamentarium, consisting of ECM degrading enzymes such as collagenase, elastase, and proteases, as well as the capacity to form ROS (37). When activated by appropriate stimuli, neutrophils induce a respiratory burst, which is marked by an increased consumption of oxygen and generation of superoxide anions, hydrogen peroxide, and hypochlorous acid (37). These free radicals are highly cytotoxic and can induce tissue injury (42). It remains unclear whether neutrophils release their cytotoxic content upon contact with BMSCs. Future studies may investigate whether inhibition of neutrophil degranulation and ROS release prevents a negative effect on the osteogenic potential of BMSC. In addition to ECM degrading enzymes and ROS, neutrophils synthesize several cytokines, such as TNF-α (43) and IL-17 (44), that have been shown to inhibit osteogenic differentiation of BMSCs (45).

Our finding that PBMCs and neutrophil transwell experiments do not induce a significant decrease in ALP activity suggests that the negative effect of neutrophils on BMSCs is not caused by depletion of nutrients from the culture medium. Figure 3C shows that BMSC numbers increase during the first 2 days when cultured in BM without neutrophils, which suggests proliferation of BMSCs. The number of BMSCs significantly decreased during the first 2 days of co-culture with neutrophils. This may be caused by inhibition of proliferation of BMSCs, neutrophil induced cell death of BMSCs or a combination of both. However, we were unable to demonstrate a decrease in BMSCs viability (Figure 3C), which makes neutrophil induced BMSC cell death *in vitro* unlikely.

Our finding that neutrophil transwell experiments did not induce a significant decrease in ALP+ cells, suggests that neutrophils need to be in proximity of BMSCs to exert their effect. It remains unclear whether neutrophils mediate their effect by binding of their cell surface receptors to BMSCs, by local release of cytokines (46) or other factors (e.g., ROS) within an immunological synapse (47). The mechanism through which neutrophils exert their effect on BMSCs may be the focus of future research.

Moreover, only little is known about whether BM, OM, or EM best represents the concentrations of growth factors present within the FH *in vivo*. We used BM in most of the co-cultures in order to minimize the amount of growth factors that could affect either neutrophils or BMSCs. It is possible that the effect of neutrophils on BMSCs disappears when fibroblast growth factor (basic fibroblast growth factor used in EM) or bone morphogenetic protein-2 (used in OM) are added to the culture medium.

It has been shown previously that mononuclear cells can stimulate osteogenic differentiation of BMSCs *in vitro* and stimulate bone regeneration (48–51). We have previously shown that—in contrast to neutrophils—monocytes/macrophages remain present within the FH during the second week of fracture healing, when BMSCs are the most prevalent cell type within the human FH (13). A recent animal study showed that induction of the regenerative M2 macrophage phenotype by interleukin 4 and 13 significantly enhanced bone formation in mice (11). M2 macrophages secrete high levels of anti-inflammatory cytokines, fibrogenic, and angiogenic factors that serve to resolve inflammation and stimulate tissue regeneration (52, 53). By contrast, M1 macrophages have a pro-inflammatory phenotype, exhibiting increased phagocytic activity and secretion of pro-inflammatory cytokines that aid in the removal of pathogens and injured tissue (54). Severe trauma induces release of different neutrophil subsets into the peripheral circulation, which includes young banded neutrophils and hypersegmented neutrophils (33). It is tempting to speculate that neutrophils can also acquire an inflammatory or regenerative phenotype that affect fracture healing differently. Future studies may focus on the role of different neutrophil subsets in fracture healing and whether trauma-induced systemic inflammation disturbs the balance between regenerative (M2) macrophages and inflammatory (M1) macrophage within the FH.

In conclusion, our data shows that human neutrophils negatively affect synthesis of mineralized ECM by BMSCs *in vitro*. Prolonged or increased influx of neutrophils into the FH after hyper-inflammatory conditions may impair fracture healing by negatively affecting ECM synthesis by BMSCs. Clarifying the underlying mechanism may contribute to development of therapies that augment bone regeneration or prevent impaired fracture healing after hyper-inflammatory conditions.

## Ethics Statement

The medical-ethical committee of the University Medical Center of Utrecht has approved isolation and use of residual samples after anonymization without informed consent. Leukocytes from peripheral blood of healthy donors were acquired after informed consent. The medical-ethical committee of the University Medical Center of Utrecht also approved isolation and use of peripheral blood of healthy donors after informed consent and anonymization.

## Author Contributions

OB designed the study, performed the main experiments and statistical analysis, and wrote the main manuscript text. MC aided in designing the study and performed experiments. JA, LK, LL, and TB aided in designing the study. All authors reviewed the manuscript.

## Conflict of Interest Statement

All authors declare that they or their institution did not receive any payment or service from third parties other than mentioned in the acknowledgments section for any aspect of the submitted work. In addition, all authors declare no patents, copyrights, (financial) relationships, or activities that may influence or give the appearance of potentially influencing what they wrote in the submitted work.

## Footnotes

^1^http://www.med.cam.ac.uk/wp-content/uploads/2016/02/SpotDetector_V4_LC06210800.pdf (Accessed: April 21, 2018).

^2^https://www.thermofisher.com/nl/en/home/brands/molecular-probes/key-molecular-probes-products/live-dead-viability-brand-page.html# (Accessed: April 21, 2018).

^3^http://www.bdbiosciences.com/ds/pm/tds/559763.pdf (Accessed: April 21, 2018).
